# Phytotoxic Activity of the Natural Compound Norharmane on Crops, Weeds and Model Plants

**DOI:** 10.3390/plants9101328

**Published:** 2020-10-09

**Authors:** David López-González, David Ledo, Luz Cabeiras-Freijanes, Mercedes Verdeguer, Manuel J. Reigosa, Adela M. Sánchez-Moreiras

**Affiliations:** 1 Department of Plant Biology and Soil Science, Faculty of Biology, University of Vigo, Campus Lagoas-Marcosende s/n, 36310 Vigo, Spain; davidledoblanco92@hotmail.com (D.L.); lcabeiras@uvigo.es (L.C.-F.); mreigosa@uvigo.es (M.J.R.); adela@uvigo.es (A.M.S.-M.); 2 CITACA, Agri-Food Research and Transfer Cluster, Campus da Auga, University of Vigo, 32004 Ourense, Spain; 3 Mediterranean Agroforestry Institute (MAI), Universitat Politècnica de València, Camino de Vera s/n, 46022 València, Spain; merversa@doctor.upv.es

**Keywords:** norharmane, phytotoxicity, *Arabidopsis thaliana*, crops, weeds, water stress

## Abstract

Norharmane is a secondary metabolite that appears in different species of land plants. In this paper, we investigated for the first time the specificity of norharmane through germination and growth tests on some crops as *Zea mays* L. (maize), *Triticum aestivum* L. (wheat), *Oryza sativa* L. (rice) and *Lactuca sativa* L. (lettuce) and weeds as *Amaranthus retroflexus* L. (amaranth), *Echinochloa crus-galli* L. (barnyard grass), *Plantago lanceolata* L. (ribwort), *Portulaca oleracea* L. (common purslane) and *Avena fatua* L. (wild oat), and its phytotoxic capacity on the metabolism of adult *Arabidopsis thaliana* L. (thale cress) by measuring chlorophyll *a* fluorescence, pigment content, total proteins, osmotic potential and morphological analysis. Norharmane had an inhibitory effect on the germination of *A. fatua* and *P. lanceolata*, and the growth of *P. oleracea*, *E. crus-galli* and *A. retroflexus*. On adult *A. thaliana* plants, the compound was more effective to watering, leading to water stress that compromised the growth of the plants and ultimately affected the photosynthetic apparatus. Therefore, this research shows that norharmane not only affects seedlings’ metabolism, but also damages the metabolism of adult plants and can be a potential model for a future bioherbicide given its specificity.

## 1. Introduction

Weeds are one of the main problems in agriculture, since they cause great losses in crop yields. Currently, commercial herbicides are based on modes of action discovered about 30 years ago and which are based on about 20 modes of action, but no new ones have recently been discovered [1]. Plants in this situation generate resistance due to some alteration in the molecular target where the herbicide acts, managing to occupy those ecological spaces provided by herbicides and finding themselves in an environment where there is no competitive pressure, which makes them more able to develop more easily [1]. In the most extreme cases, these species can generate resistance to multiple modes of action. Due to their structural diversity, natural compounds are a good source for new modes of action in the development of new herbicides. In fact, there are various natural compounds of microbial or plant origin that show molecular targets different from those of commercial herbicides [2], which suggests that there are many more potential sites of action for herbicides than are used today. Dayan et al. [2] described a number of natural phytotoxin target sites that are different to those of commercial herbicides, and that can be as active as synthetic herbicides, but perhaps due to their complexity or to their physicochemical properties [1], bioherbicides based on these phytotoxins have not been largely developed.

However, the great structural diversity of these metabolites and its natural origin makes them a priori more environmentally friendly and a possible alternative to the resistance issue [3]. From these natural products it would be possible to obtain a bioherbicide, defined as a product of natural origin (live organisms or products derived from them) to be used to control weeds [4]. An example of a bioherbicide would be Beloukha^®^, authorized since 2015 in Europe and since 2016/2017 in the U.S. and Japan, and produced through natural extraction of rapeseed oil, obtaining nonanoic and pelargonic acid [5]. Generally, these compounds can have an effect on photosynthesis, hormonal balance, nutrient uptake, formation of reactive oxygen species (ROS) and germination and growth of weeds [6].

Moreover, the fact that the herbicide is effective against germination (pre-emergence) or growth (post-emergence) of weeds is an important factor, since it is part of the specificity of the compound. Therefore, both measurements must be done in the phytotoxic study of a compound. Currently, there are several examples of synthetic herbicides that are applied before germination, such as xadiargyl, acetachlor, pendimethalin and after germination, such as glyphosate, imazethapyr, formesafen or pyrazosulfuron [7,8,9].

**Figure 1:**
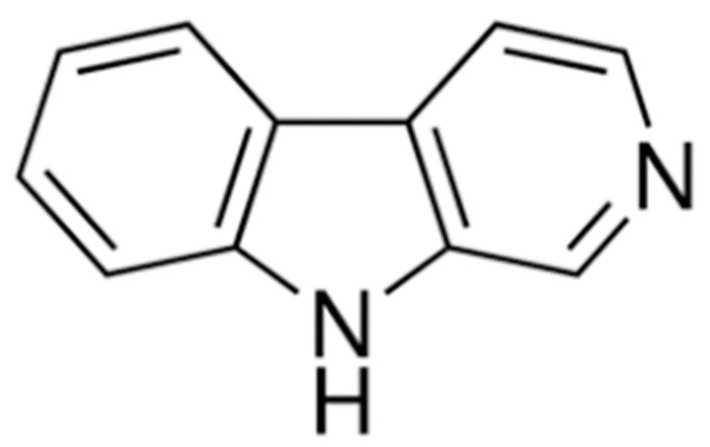
Norharmane chemical structure.

Norharmane (9H-pyrido (3,4-b) indole, NOR; Figure 1) is a secondary plant metabolite that belongs to the β-carbolines group, an indole alkaloids group [10]. It can be naturally found in plants of the Gramineae, Sapotaceae and Zygophyllaceae families. The compound was isolated for the first time from the *Peganum harmala* plant [11]. It is not found exclusively in land plants, but it can also be found in marine organisms such as *Noctiluca miliaris* or *Nodularia harveyana* [12]. As a matter of interest, these β-carbolines can be found in roasted coffee beans and chicory [13]. Epidemiological studies showed that there is a negative correlation between drinking coffee and the incidence of Parkinson’s disease [14] as these compounds would act as neuromodulators, inhibiting the monoamine oxidase (MAO) enzyme [15]. In addition, NOR has a number of pharmacological effects, such as the increase of insulin secretion in isolated isles of Langerhans [16], and being a compound that has a comutagenic activity [17], making NOR a compound of toxicological importance.

Regarding the phytotoxic capacity of the compound, it has also been discovered that NOR has effects on the metabolism of certain plant species. NOR extracts obtained through the *Synechocystis aquatilis* cyanobacteria showed negative effects on other species of cyanobacteria, such as *Microcystis aeruginosa* and *Oscillatoria limnetica*, and on two species of green algae, such as *Chlorella vulgaris* and *Ulothrix* sp. [18]. Recently, NOR has been found to have phytotoxic effects on *A. thaliana* seedlings, inhibiting their growth by altering the polar auxin transport, as a result of inhibiting the proteins PIN2, PIN3 and PIN7 that are involved in the transport of auxin [19]. The inhibition of such PIN proteins resulted in an increase of secondary and adventitious roots, the alteration of the cell division, the presence of incomplete walls and the decrease of root length.

However, this prior research used *A. thaliana* as a model, which is a very sensitive species to phytotoxicity. Therefore, weeds and crops bioassays were developed to verify whether NOR has phytotoxic effects on different crop species and their associated weeds and to confirm the presence of specificity for their use in certain agroecosystems. Additionally, since the research conducted so far has been conducted on the seedling metabolism (much more sensitive to phytotoxicity by definition) it is necessary to further research the phytotoxic effect that this compound may have on the metabolism of adult plants, using as a model the species *A. thaliana* and using NOR in two different ways, through irrigation and pulverization, to find out if the compound is more effective in contact with the leaves or absorbed by the root.

## 2. Results

### 2.1. Effects of Norharmane on the Germination and Growth of Weeds and Crops

As observed on Table 1, NOR affected very lightly the germination process of the weeds tested, both in terms of the total germination rate (G_T_) and the germination speed (S, AS). Only the effect on *Plantago lanceolata* and *A. fatua* was relevant in the weeds group. NOR reduced significantly the total germination (G_T_), and induced a delay in the germination (S and AS) of *P. lanceolata* at 800 µM with G_T_, S and AS values of 27.1% with respect to the control, 1 and 1.6 respectively. As for *A. fatua*, the concentration 800 µM respectively, caused a significant reduction, with a decrease of G_T_ of 57.0%, which was the only concentration tested that also caused a delay in germination with a S value of 1.88 and an AS value of 6.33, both values are half that in the control.

As for the crops group, NOR did not cause stimulatory or inhibitory effects on the germination of *O. sativa*, but for *Z. mays* germination was inhibited at the highest concentration (800 µM) of the compound, with values of G_T_, S and AS of 82.4%, 6% and 14.0% respectively. *T. aestivum* germination was also slightly delayed, but just at the highest concentration tested, with values of S and AS of 9.36 and 21.6, respectively. For *L. sativa*, which was the most sensitive to NOR, as both its total germination and germination speed were affected from 200 µM NOR, with values of G_T_ of 71.6%, S of 1.64% and AS of 4.90%.

By contrast, as observed on Table 2, NOR had a significantly stronger effect on the root growth of the species tested. This process was especially affected in the weeds group, as their growth was strongly inhibited after the treatment with NOR. The most affected weeds were *P. lanceolata* and *E. crus-galli*, whose growth was inhibited already with the smallest concentration of NOR (50 µM) showing a root length of 75.0% and 85.0% respectively. *P. oleracea* was a particular case because its growth was inhibited with concentrations of 400 and 800 µM (values of 57.1% and 9.85% respectively), but it was stimulated at the lowest concentrations tested, 50 and 100 µM (values of 154% and 142% respectively), a clear example of the hormesis phenomenon, by which a substance can have stimulatory effects at low doses but be inhibitory at high doses [20].

As for the crops group, NOR inhibited the growth of *T. aestivum* at the concentrations of 400 and 800 µM (74.9% and 51.3% with respect to the control respectively) and *L. sativa* at concentrations of 400 and 800 µM (values of 67.3% and 38.8% respectively), which was highly inhibited, although its growth was stimulated at low concentrations of the compound. It is important to mention maize’s reaction to NOR, as its growth was not affected by any of the NOR concentrations tested, and it was even stimulated in some of the highest concentrations such as 200 and 400 µM (values of root length of 110% and 111% respectively). The behavior of rice is also interesting, as it was subjected to a slight inhibition in some NOR concentrations, which was never over 15% for any of the concentrations tested.

**Figure 2:**
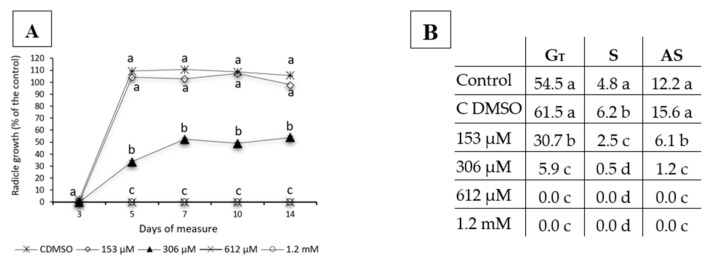
(**A**) Radicle growth and (**B**) germination indices (G_T_, S and AS) for *Erigeron bonariensis* seeds after treatment with 0 (control), 0 + DMSO (control with DMSO), 153 µM, 306 µM, 612 µM and 1.2 mM concentrations of norharmane (NOR). Germination and growth were recorded after 3, 5, 7, 10 and 14 days to monitor both processes. Letters indicate statistical differences among treatments for germination (**A**) or radicle growth (**B**) at *p* < 0.05. One letter for more than one treatment means that all the treatments have the same significance.

Regarding monitoring of germination and growth of *Erigeron bonariensis* L. (syn. *Conyza bonariensis*), we can see in Figure 2 that all the concentrations tested induced the inhibition of the total germination and germination speed (Figure 2B), at over 50.0% for the lowest concentration tested (153 µM) and at 90.0% for the 306 µM concentration of NOR, when compared to the control. The effect on germination was so intense that none of the seeds treated with the two highest concentrations tested (612 µM and 1.2 mM) germinated during the 14 days that the experiment lasted, and no recovery of the germination process was observed. As for growth (Figure 2A), although the effect observed was not strong, with no significant differences in seedlings that grew at a concentration of 153 µM, there was a clearly stable effect during the 14 days for the highest concentration tested, which did not induce total inhibition of germination (306 µM).

### 2.2. Effect of Norharmane on A. thaliana Adult Plants

#### 2.2.1. Morphological Study

**Figure 3:**
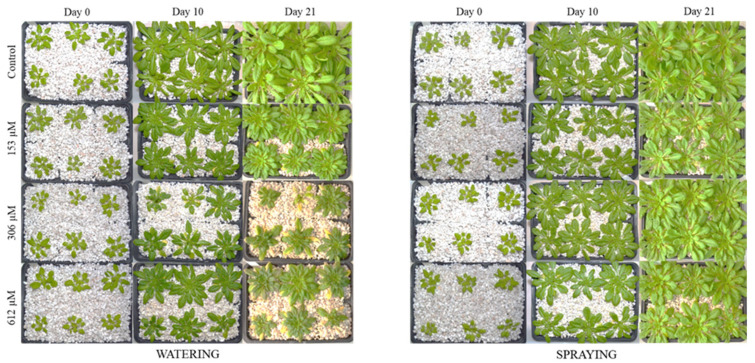
Adult *A. thaliana* plants watered and sprayed with different norharmane concentrations (0, 153, 306 and 612 µM) after 0, 10 and 21 days of treatment.

After the 21-day phytotoxicity bioassay, the *A. thaliana* plants watered with NOR suffered important morphological changes. As observed in Figure 3, the development of plants was highly reduced after watering with all NOR concentrations, although it was especially significant in plants treated with 306 and 612 µM. The effects of NOR were observed not only in the strong reduction of the fully grown plant’s size, but also in the size of the leaves, which were smaller and less developed. Moreover, the leaves of plants watered with NOR also had some yellowish, brownish and purple areas, particularly visible during the last days of treatment. The effects on the leaves were much greater on plants that were treated with the highest concentrations (306 and 612 µM).

**Figure 4:**
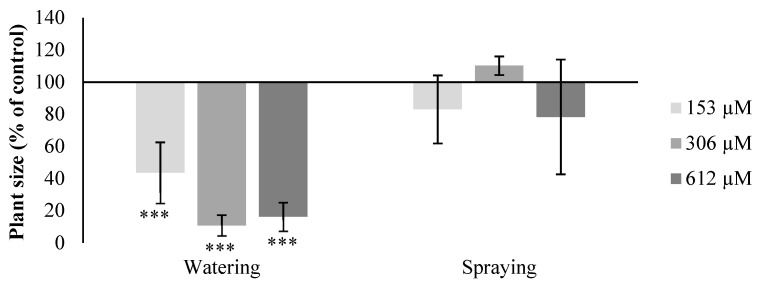
Plant size in the percentage with respect to the control of *A. thaliana* watered and sprayed with different concentrations of norharmane (0, 153, 306 and 612 µM) for 21 days. Asterisks indicate significant differences from controls (*** *p* ≤ 0.001).

However, plants pulverized with NOR did not show apparent morphological changes among the different concentrations tested, and just a slight reduction of the fully grown plant’s size was noted with the highest concentration (Figure 3). If we compare the pulverization with the irrigation treatments at the same concentration, for example 306 µM, we observed great homogeneity in the size of the plants that were pulverized, very similar to the control treatment, whereas the NOR-watered plants registered a decrease of their morphological variety and changes in color compared to the control plants (Figure 3 and Figure 4).

#### 2.2.2. Leaf Area

Regarding the leaf area, the plants watered with NOR showed a significant decrease compared to the control sample, of up to 57%, 90% and 84% in plants treated with concentrations of 153, 306 and 612 µM, respectively, although the last two did not register significant differences between them as the NOR treatment had a strong effect in the development of the fully grown plant, inhibiting it almost completely (Figure 3 and Figure 4). No significant changes were observed in the leaf area of the plants treated through pulverization.

#### 2.2.3. Colorimetric Analysis

**Figure 5:**
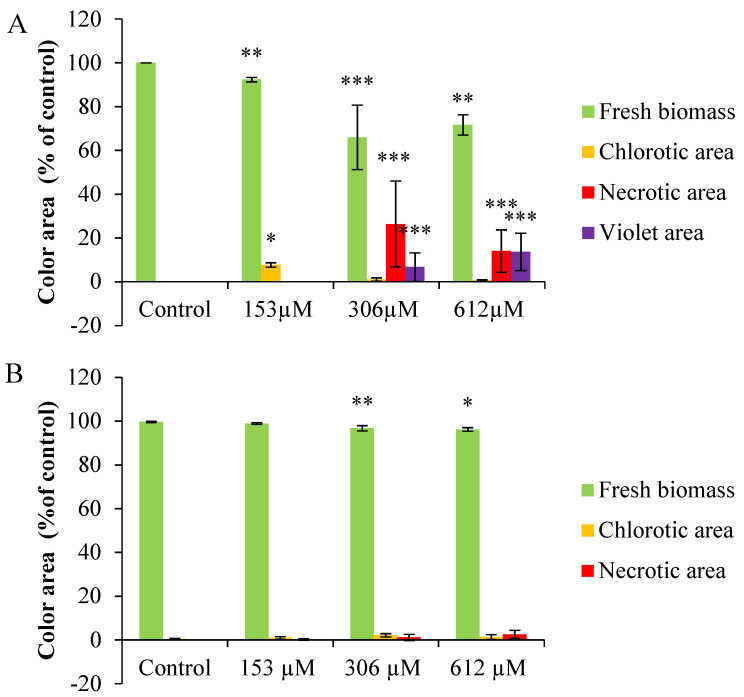
Colorimetric analyses of the leaf area of *A. thaliana* plants watered (**A**) and sprayed (**B**) with different concentrations of norharmane (0, 153, 306 and 612 µM) for 21 days. Asterisks indicate significant differences compared to the control (* *p* ≤ 0.05, ** *p* ≤ 0.01, *** *p* ≤ 0.001). Data are given in percentage with respect to the control.

If the NOR-watered plants are observed in detail, chlorotic (yellowish) and violet areas, especially noticeable on the underside of the leaves, can be found. In fact, the colorimetric analysis of the leaves of plants watered with NOR showed significant differences in the fresh biomass at all concentrations, with a decrease of fresh biomass and an increase of chlorotic areas (Figure 5A). For the plants watered with 306 and 612 µM NOR, the reduction of fresh biomass (65% and 71%, respectively) was related to an increase of the necrotic (26% and 14%, respectively) and purple areas (6% and 13%, respectively). By contrast, the colorimetric analysis of *A. thaliana* leaves pulverized with NOR (Figure 5B), showed only a slight decrease of fresh biomass (green color) at the highest concentrations tested (306 and 612 µM) and it was linked to a small increase of chlorotic (yellowish) and necrotic (brown) areas.

#### 2.2.4. Anthocyanin Content

**Figure 6:**
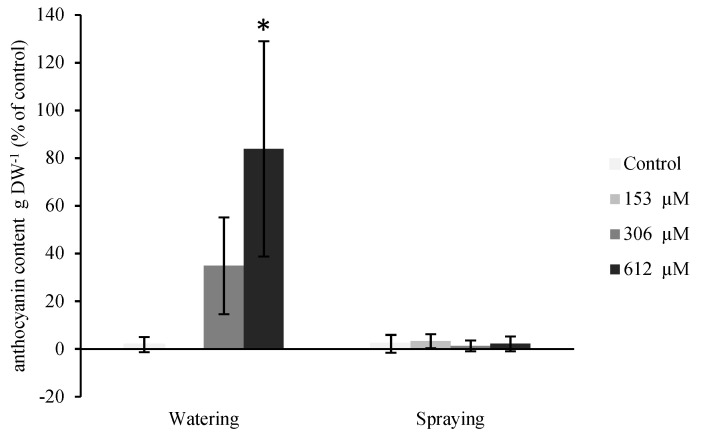
Anthocyanin content per gram of dry weight (DW) of *A. thaliana* plants watered and sprayed with different concentrations of norharmane (0, 153, 306 and 612 µM) for 21 days. Asterisks indicate significant differences compared to the control (* *p* ≤ 0.05). Data are given in percentage with respect to the control.

As the violet areas are normally associated to an accumulation of anthocyanins, this pigment was quantified in the plants treated with NOR (Figure 6). As with the leaf area, the plants that were pulverized with norharmane did not show significant differences between the control sample and the treatments, whereas the plants watered with 306 and 612 µM NOR considerably increased their content in anthocyanins, obtaining significant differences in the plants watered with the highest concentration (612 µM), which increased their content in 83.9% when compared with the control plants. Although the plants watered with 306 µM NOR did not show significant differences, given the high variation of the data, an increasing trend could be observed in the anthocyanin content, with an increase of 34.9% compared to the control plants.

#### 2.2.5. Chlorophyll and Carotene Content

**Figure 7:**
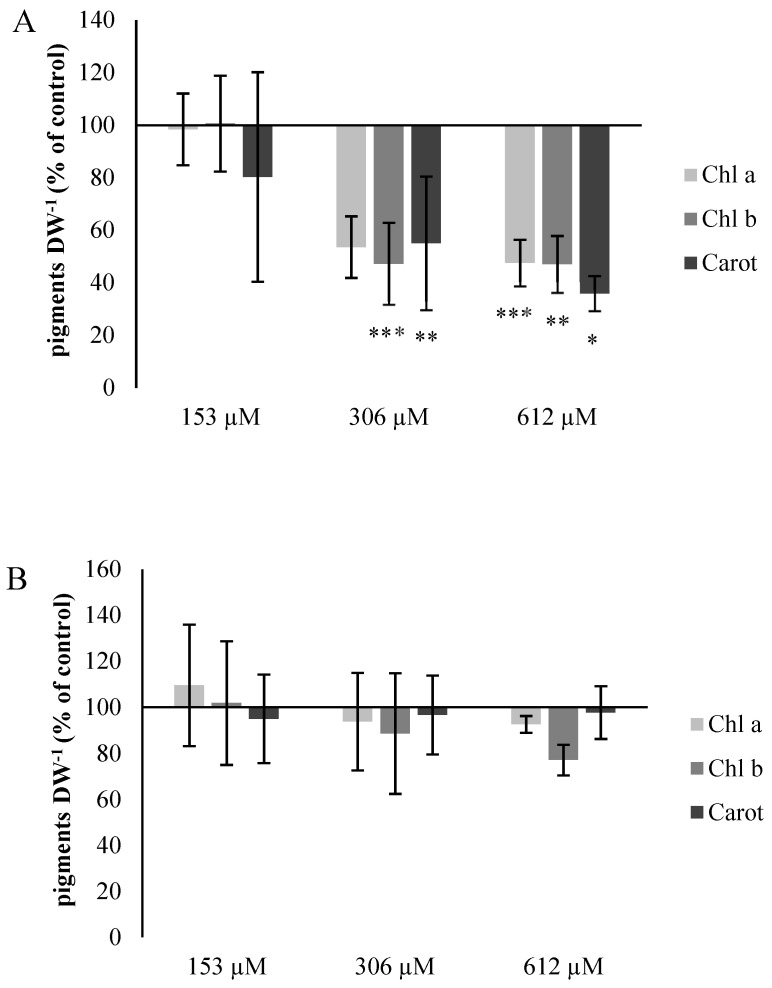
Pigments (chlorophyll *a*, chlorophyll *b* and carotenoids) content (percentage of control) of *A. thaliana* plants watered (**A**) and sprayed (**B**) with different concentrations of norharmane (0, 153, 306 and 612 µM) for 21 days. Asterisks indicate significant differences compared to the control (* *p* ≤ 0.05, ** *p* ≤ 0.01, *** *p* ≤ 0.001). Data are given in a percentage with respect to the control.

The presence of chlorotic and violet areas in the plants watered with NOR was related to a strong effect in the content of pigments per gram of dry weight (DW) expressed as a percentage compared to the control plants (Figure 7A). This value was significantly reduced for the three types of pigments in plants irrigated with 306 and 612 µM NOR. The values of the pigments content were 53% for Chl *a*, 47% for Chl *b* and 55% for carotenoids in plants watered with the 306 µM concentration compared to the control sample, whereas the results for the plants watered with 612 µM NOR were 47% for Chl *a*, 47% for Chl *b* and 35% for carotenoids compared to the control sample. By contrast, in the case of pulverized plants (Figure 7B), no significant changes were found.

#### 2.2.6. Chlorophyll *a* Fluorescence Measurement

**Figure 8:**
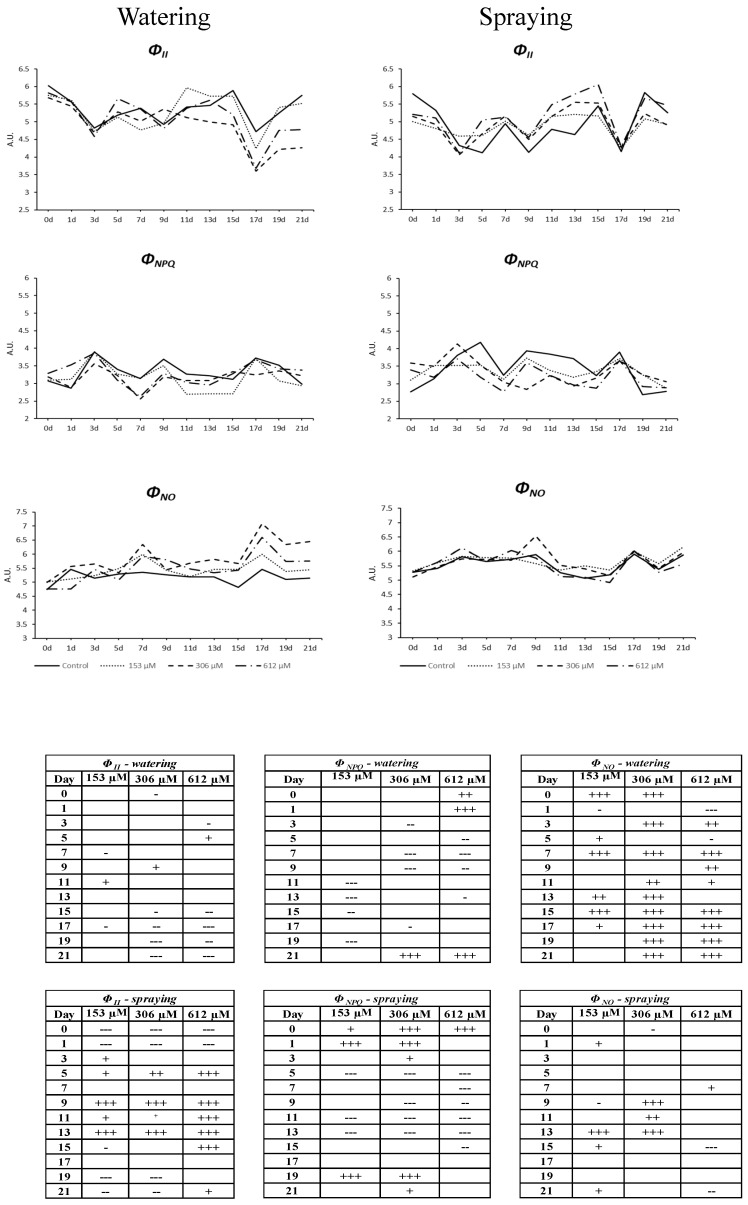
Mean values, expressed in arbitrary units (AU), of Φ_II_, Φ_NPQ_ and Φ_NO_ from *A. thaliana* adult plants watered or sprayed for 21 days with different concentrations of norharmane (0, 153, 306 and 612 µM). Tables show the statistical significance compared to the control on each day and treatment (+, stimulatory difference; -, inhibitory difference; + or -, *p* ≤ 0.05, ++ or --, *p* ≤ 0.01, +++ or --- *p* ≤ 0.001).

**Figure 9:**
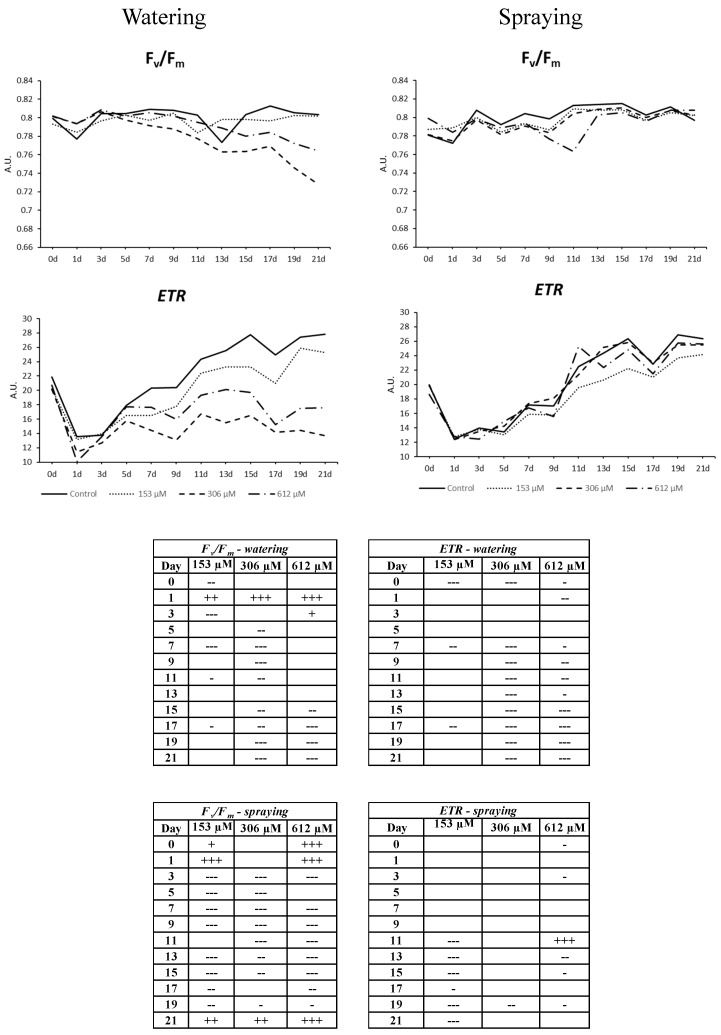
Mean values, expressed in arbitrary units (AU), of F*v*/F*m* and electron transport rate (ETR) in *A. thaliana* adult plants watered or sprayed for 21 days with different concentrations of norharmane (0, 153, 306 and 612 µM). Tables show the statistical significance compared to the control on each day and treatment (+, stimulatory difference; -, inhibitory difference; + or -, *p* ≤ 0.05, ++ or --, *p* ≤ 0.01, +++ or --- *p* ≤ 0.001).

This effect on photosynthetic pigments was also linked to the alteration found for the parameters related to photosynthetic efficiency, especially the non-regulated energy emission and the electron transport rate (Figure 8 and Figure 9). Regarding plants pulverized with NOR, only a slight increase of photochemical activity (Φ_II_) was observed during the central days of the experiment, with the subsequent decrease of dissipation as heat (Figure 8), although no significant differences were observed in those parameters related to damage in the photochemical stage, such as the intrinsic efficiency of PSII (F*v*/F*m*) or the increase in fluorescence emission (Φ_NO_). By contrast, the NOR-watered plants showed a clear effect on fluorescence emission and photochemical efficiency, which supports the results obtained in the colorimetric and photosynthetic pigment analyses.

Therefore, the parameter Φ_NO_, non-regulated dissipation of energy (mainly fluorescence) significantly increased in all plants watered with NOR as of the fifth day, particularly in the highest concentrations tested (306 and 612 µM, Figure 8), where Φ_NO_ reached peak values and maintained them until the end of the experiment. In contrast, although the plants watered with the lowest concentration (153 µM) showed an increase of their fluorescence values during the central days of the experiment, they got stabilized and the levels were similar to the control levels by the end of the experiment. Other parameters, such as the efficiency of photosystem II (Φ_II_), the electron transport rate (ETR) or the maximum efficiency of PSII (F*v*/F*m*, Figure 9) showed also values that were similar to the control during the last days of the treatment for plants watered with the lowest concentration. However, the increase of fluorescence emission in plants watered with the highest concentrations of NOR was related to a decrease of the electron transport rate (ETR) throughout the experiment (Figure 9), and a decrease of the efficiency of photosystem II (Φ_II_) from day 15, being the plants watered with 306 and 612 µM NOR the most inhibited. 

Lastly, the strong effect on the electron transport rate along the experiment resulted in a significant decrease of the maximum efficiency of PSII (F*v*/F*m*), especially strong from day 15 of the experiment (Figure 9). By contrast, the regulated dissipation of energy as heat (Φ_NPQ_) did not show relevant differences between the NOR-watered and the control plants, although significant decreases were registered for some periods and concentrations (Figure 8).

#### 2.2.7. Other Measures

**Figure 10:**
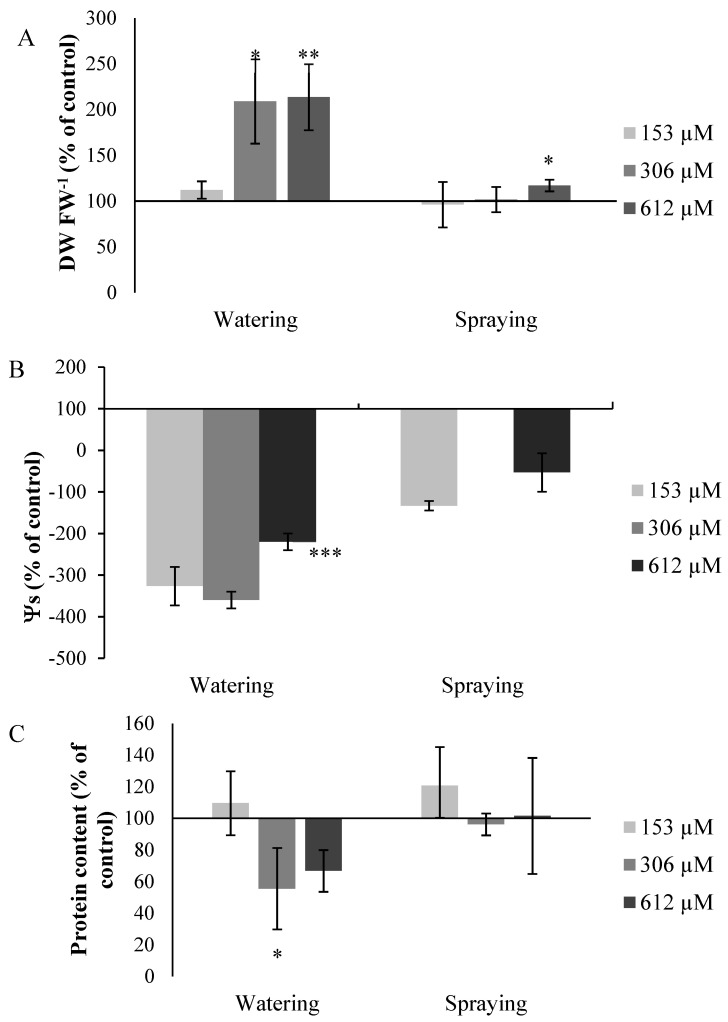
Values for the DW FW^−1^ ratio (**A**), osmotic potential, Ψs (**B**) and protein content (**C**) of *A. thaliana* plants watered or sprayed with different concentrations of norharmane (0, 153, 306 and 612 µM) for 21 days. Asterisks indicate significant differences compared to the control (* *p* ≤ 0.05, ** *p* ≤ 0.01, *** *p* ≤ 0.001). Data are given in a percentage with respect to the control.

Regarding the measures related to the water level of the plants treated, such as the dry weight/fresh weight ratio (DW/FW) and the osmotic potential (Ψs), the plants pulverized with NOR also did not register significant differences in the parameters, whereas the plants watered with the compound registered significant differences that were relevant for the water level of the plants treated. The DW/FW ratio significantly increased in all the plants watered with NOR (Figure 10A), by 112% for the plants treated with 153 µM, by 208% for those treated with 306 µM, and by 213% for those treated with 612 µM NOR. As for the osmotic potential, the plants watered with NOR showed a decrease of 326%, 360% and 220% for the plant leaves watered with concentrations of 153, 306 and 612 µM of NOR, respectively (Figure 10B). Lastly, it should be noted that the concentration of total proteins in the plants treated decreased in the plants watered with NOR, but the decrease was only significant in the treatment of 306 µM, with a decrease of 45% (Figure 10C). There were no significant changes in the plants treated through pulverization. 

## 3. Discussion

As suggested by the weed and crop bioassays, norharmane did not affect germination in the same way it affected root growth, and neither affected equally all the species tested, showing specificity for certain agricultural systems. It is frequent to observe after treatment with biomolecules or plant extracts how they act differently depending on the species and in a dose-response manner [21]. In general, NOR seems to affect the growth (root length) more than the germination of the species, affecting weeds more negatively than crops growth, with some exceptions, such as lettuce, which is very sensitive to this compound, or wheat, which is inhibited at high doses of the compound.

The use of NOR in maize agroecosystems is especially interesting, as this crop was not affected by the compound at any of the doses tested, and its growth was actually stimulated at some of the highest concentrations tested (200 and 400 µM). However, the development of weeds associated to this crop, such as *E. crus-galli*, *P. oleracea* and *A. retroflexus* [22] was strongly inhibited in the presence of NOR, with inhibitions of the root growth of up to 70.0% for *P. oleracea* or 50.0% for *E. crus-galli* and *A. retroflexus*, inhibitions that would not allow for the complete eradication of weeds, but would provide an important advantage to the crop in terms of competition, preserving at the same time the biodiversity of the ecosystem. Increasing biodiversity normally increases production too, providing also different socioeconomic, nutritional and environmental benefits. [23]. Using a compound to control weeds, which reduces weed resistance and stimulates the growth of the crops, can be highly beneficial to maintain such biodiversity.

As for the rice, significant inhibitions in the presence of NOR were under 13.0% compared to the control, so that can be considered non relevant for the development of the crop. By contrast, the weeds associated to rice, such as *E. crus-galli* and *P. oleracea*, were very sensitive to NOR, registering a 70.0% decrease of the growth of this species. Moreover, the fact that NOR is able to reduce the growth of *E. crus-galli* makes it a particularly interesting compound as a potential bioherbicide, as this species is not only considered one of the most important weeds worldwide that is especially resistant to traditional herbicides [24], but it is also one of the most harmful weeds for the development of rice, competing with it for essential factors and even showing allelopathic activity against rice [25].

Finally, NOR has a strong effect on the growth of *A. fatua* and *P. lanceolata*. The growth of these weeds was also negatively affected in comparison to other natural compounds such as citral [26] or trans-chalcone [27], although NOR resulted in being more efficient in terms of inhibition of the root growth. Nonetheless, the compound seems to also affect wheat growth, a crop associated to those two weeds, reaching an inhibition of up to 50%, so the use in these agroecosystems is not highly advisable. By contrast, given the strong inhibition of *P. lanceolata* in NOR treatments and given the regular presence of this species along roadsides and in the intercropping spaces, NOR can be considered an interesting compound to control *P. lanceolata* in those conditions.

Another way to control weeds is through avoiding seed germination. NOR does not seem to have a very strong effect on germination, only affecting relevantly total germination and germination speed of *A. fatua* and *P. lanceolata*. It is very common that species respond differently to the tested compounds, as the characteristics of the seeds (i.e., size, permeability or absorption mechanisms, among others) play a very important role in sensitivity [28]. Contrarily to root growth (post-emergence), where the use of NOR in wheat agroecosystems was not recommended, it would be interesting to use NOR in the pre-emergence control of weeds for the agroecosystems *T. aestivum*-*A. fatua* or *T. aestivum*-*P. lanceolata*, as NOR does not affect total germination, but it does affect the germination of such weeds (up to 50.0% for *A. fatua*), so the crop may obtain an important ecological advantage against such weeds, as the total number of weeds germinated and the resistance of future plants is reduced in the early stages [29], and it can even be used to reduce the seed bank of these weeds in a fallow land before planting the crop chosen and reducing the pressure of weeds on it. Previous knowledge and control of the seed bank of the soil will allow a more rational use of herbicides/bioherbicides during the development of the crop [30].

Although these tests show specificity of NOR for different species, it must be taken into account that the phytotoxicity of a natural compound can be affected by environmental conditions (light, temperature, etc.) [31,32,33]. This point must be considered when analyzing the results. Therefore, in the trial, the optimal conditions for each species have been chosen, so that we are measuring each species in a situation of humidity, temperature and photoperiod that would potentially occur in the cultivation land in which the norharmane could act.

On the other hand, the monitoring of NOR on the growth of the weed species *E. bonariensis*, considered an invasive weed in Spain [34] and is important as an invasive species in maize [35] and wheat [36] fields, showed that the effect of this compound on the growth happens two days after germination and remains throughout time without almost no variations in the intensity of the inhibition produced, which was close to 60.0% during the 14 days that the plants treated with the 306 µM NOR concentration were measured. Similarly, the two highest concentrations tested (612 µM and 1.2 mM) did show a total inhibition of germination, and no recovery was observed during the 14 days of measurement, which makes the effectivity of this compound an advantage, given the stability of its effects on *E. bonariensis* weed, during the pre and the post-emergence. Considering that *E. bonariensis* is a harmful weed globally and it reproduces by releasing a massive amount of seeds to the environment, the control and delay of the germination of the seeds with concentrations as low as 153 µM NOR would help control this weed in rural and urban areas.

Regarding the effects of NOR on the metabolism of adult plants, we can confirm according to our results that this secondary metabolite causes morphological and physiological alterations in the plants treated, which are different depending on the method used to apply the compound (irrigation or spraying). Whereas plants pulverized with NOR registered only a slight decrease in size, together with a decrease of healthy areas and a slight increase of yellowish and brown areas in the leaves, the plants watered with NOR showed clear symptoms that the compound was seriously affecting their development, with a strong decrease in the size of rosettes and healthy green areas in the leaves, and an increase of yellowish (chlorotic), brown (necrotic) and even violet (accumulation of anthocyanins) areas. These results led us to believe that NOR is more effective when it is absorbed by the roots. The translocation of the compound, or the triggering of signals in the roots that exert their effect in the aerial part makes this form of application more effective.

The decrease in size of the plants watered with NOR, the reduction of the leaf area, the increase of the DW/FW ratio and the decrease of Ψs, which becomes increasingly negative for the concentrations tested, seem to suggest that the water status of the plants is being altered after irrigation with NOR. The water deficit has a strong effect on cell growth due to the decrease of the turgor pressure within the cells, and cell elongation can be affected by the interruption of the water flow from the xylem, which leads to the formation of smaller plants with a smaller leaf area [37]. A similar pattern was observed in *A. thaliana* plants treated with the secondary metabolite *trans*-chalcone [27]. In water stress situations it is common to observe a decrease in Ψs [38], as observed in plants watered with NOR, which would support the idea that the compound causes water stress in the plant. There are several natural compounds that induce the decrease of the osmotic potential, as demonstrated for p-hydroxybenzoic acid when applied on *Dactylis glomerata* plants [39].

Another symptom of water stress is the appearance of violet areas in the leaves, which are related to an increase of the anthocyanin content, whose synthesis is promoted in situations of drought stress and plays an important role in photoprotection, by selecting the visible light and/or suppressing reactive oxygen species (ROS) through its strong antioxidant capacity [40]. As verified when quantifying this pigment, the plants watered with NOR registered a significant increase of anthocyanins, which supports the idea that NOR is generating water stress in those plants.

When water stress occurs it is also frequent to observe a reduction of chlorophyll content. Ozfidan et al. [41] observed that the content in total chlorophylls (chl *a* + *b*) was reduced in *A. thaliana* plants subjected to drought stress by polyethylene glycol. This decrease in the chlorophyll content can be due to a decrease of the synthesis of the pigments or an increase of their degradation. However, during water stress, it is common that this decrease is caused by increased degradation [42], which could be the case for plants watered with NOR.

As expected, the effect observed in the chlorophylls content was related to an alteration of the photosynthetic capacity of the NOR-watered plants, as indicated by the analyzed parameters regarding chlorophyll *a* fluorescence. A decrease of chlorophylls means that the photosystems have a reduced absorption capacity and, as a result, a decrease of the photosynthetic capacity. The parameter F*v*/F*m* (intrinsic efficiency of PSII) reached values under 0.80 (reference values for healthy adult plants) [43], which indicates that the plant’s physiological state is altered and that a situation of photoinhibition or PSII activity reduction is taking place [43]. Decreases of F*v*/F*m*, as found in this paper, could suggest the presence of physical damage at the antenna system of PSII caused by NOR, as previously observed by Beninger et al. [44] for the phenolic compound chlorogenic acid and for the flavanone eriodyctiol, and by Graña et al. [26] for the monoterpenoid citral. A protection mechanism against the oxidative stress in the PSII is the regulated energy dissipation as heat (Φ_NPQ_), in which xanthophylls, located in the LHC antenna complex, play an important role [42]. The low carotenoid content observed in plants treated with NOR could be related to the fact that the Φ_NPQ_ values are stable throughout the 21 days of the test, which would indicate that the plant is unable to increase the energy dissipation in a regular manner and does it in a non-regulated manner through fluorescence, as reflected by the increase of Φ_NO_. [45] The increase of Φ_NO_ would cause the loss of the photosynthetic efficiency of PSII, as reflected by the decrease of Φ_II_ values. This behavior of the photosynthetic apparatus is similar to the one observed with the commercial herbicide Diuron [46], but it is also similar to the behavior of other plant metabolites such as 2-3H-Benzoxazolinone [47].

In a water stress situation, the first thing that the plant does is closing the stomata to avoid water loss, which causes a decrease in the fixation of CO_2_ by the photosynthetic cells and an acceleration of the senescence processes [48]. The imbalance between energy captured and energy used can produce damages in the electron transport chain, as observed in the early decrease of ETR in plants treated with NOR, which happens before the decrease of Φ_II_ and F*v*/F*m* and the increase of fluorescence emission (Φ_NO_).

All these data suggest that the plants watered with NOR are going through a water stress situation that would develop early senescence events, causing alterations in the biochemical phase of the photosynthesis due to the inability to efficiently fix CO_2_, which would cause a decrease in the ETR parameter. The excess of electrons, which cannot reach their final acceptors, would produce an overexcitation state, which would cause damages in the long term in the photochemical phase of the photosynthesis, as the plant is unable to promote regulated mechanisms to dissipate the excess energy, probably generating an oxidative stress through ROS production, which could be responsible for the decrease of pigments and the appearance of chlorotic and necrotic areas in the plants treated.

The alteration of the water status observed in NOR-irrigated plants could be caused by the fact that the compound alters the water in the plant initiating the ABA signaling cascade or the fact that the compound imitates the effect of ABA in the plants watered, as it has been previously suggested for other natural compounds [26].

## 4. Conclusions

In conclusion, NOR is more effective in the metabolism of *A. thaliana* adult plants when it is applied by irrigation, with signs indicative of water stress signaling pathways in the plant, whether by induction of the ABA synthesis or by NOR mimicking to this plant hormone. The reported sudden stop of the growth of the aerial part, the reduction of the leaf area to minimize the photosynthetic area or the closure of the stomata, with the subsequent decrease of CO_2_ absorption, would affect the biochemical phase of the photosynthesis, inducing subsequent damages in the photochemical phase, and causing the observed decrease of the photosynthetic capacity and alterations in the photosynthetic apparatus.

The strong effects observed in the plants watered with NOR confirm the bioherbicide potential of the compound, suggesting that it would be valuable to control weeds in the seedling and adult plant phases. Therefore, its use as a model for future pre-emergence bioherbicide is particularly interesting for agroecosystems *T. aestivumA. fatua* or *T. aestivum-P. lanceolata*, and as post-emergence bioherbicide for agroecosystems *Z. mays-E. crus-galli*, *Z. mays-P. oleracea* and *Z. mays-A. retroflexus*, and agroecosystems *O. sativa-E. crus-galli* and *O. sativa-P. oleracea*. On the other hand, NOR’s capacity to effectively control the growth of *A. thaliana* adult plants opens a new research and study field for the control of weeds in later vegetative stages.

## 5. Materials and Methods

### 5.1. Germination and Growth Tests

#### 5.1.1. Screening of Crops and Weeds

The secondary metabolite norharmane (NOR) was tested on different crop species and weeds associated with them in pre and post-emergence bioassays, according to Graña et al. [26] with the goal of establishing in which crop-weed system NOR could be used to control weeds. The crop species tested were *Zea mays* L. (maize), *Triticum aestivum* L. (wheat), *Oryza sativa* L. (rice) and *Lactuca sativa* L. (lettuce). Among the weeds associated with these crops, the following were selected: *Amaranthus retroflexus* L. (amaranth), *Echinochloa crus-galli* L. (barnyard grass), *Plantago lanceolata* L. (ribwort), *Portulaca oleracea* L. (common purslane) and *Avena fatua* L. (wild oat). Table 3 provides a summary of the weeds tested and the crops that they are associated with.

The seeds, previously sterilized with 1% NaOCl for 10 min and subsequently washed with tap water, were sown in Petri dishes of 9 cm diameter (14 cm for maize) on Whatman grade 3MM paper soaked with different norharmane (NOR; norharmane crystalline, Sigma-Aldrich, Saint Louis, MO, USA) solutions (0, 50, 100, 200, 400 and 800 µM), using 5 replicates per treatment. The pH was adjusted to 6.0 to avoid non-desired pH related effects. The volume of solution used depended on the species, using 11 mL of solution per dish for maize, 6 mL for wheat and 4 mL for the other species. The number of seeds per dish also varied depending on the seed size; 25 seeds were used for species of a greater size and 50 for species of a smaller size. After the seeds were sown in the dishes, they were placed in a growth chamber under conditions of a controlled temperature, humidity and a photoperiod according to each species: 16/8 h light/darkness at 26/20 °C for *L. sativa*; 8/16 h light/darkness at 22 °C for *P. lanceolata*; 16/8 h light/darkness at 16/26 °C for *P. oleracea* and *E. crus-galli*; 27 °C and complete darkness for *Z. mays* and *O. sativa*; 16/8 h light/darkness at 16/26 °C for *T. aestivum* and *A. fatua* and 35 °C and constant darkness for *A. retroflexus.*

The number of sprouted seeds was counted, and a seed was considered to have germinated when the radicle emerged 1–2 mm from the seed coat. The sprouted seeds were counted as follows: every 4 h during a 48 h period for wheat, every 3 h during a 24 h period for lettuce, every 6 h during a 48 h period for corn, every 2 h during a 10 h period for rice, every 2 h during a 22 h period for amaranth, every 12 h during a 72 h period for wild oat, every 24 h during a 168 h period for the common purslane, every 12 h during a 60 h period for ribwort and every 4 h during a 16 h period for barnyard grass.

After the germination bioassay was completed, different parameters related to the kinetics of germination were calculated in order to determine the germination speed and ratio according to Chiapusio et al. [49]:

1. Total germination (G_T_): *N_T_/N × 100*, where *T* indicates the time of the latest observation and *N* represents the total number of seeds sown.
2. Germination speed (S): the number of seeds that germinated between two measurement time points, and it was calculated as follows: *n_1_ + (1/2) × n_2_ + (1/3) × n_3_ +…+(1/T) × n_T_, where n_t_ = (N_t_ − N_t−1_)/N_T_* (with *N_0_* = 0) is the number of seeds that germinated between times *t^−1^* and *t*; it is expressed as a proportion of all the seeds that germinated. This parameter indicates the number of germinated seeds between two measurement times.
3. Accumulative germination speed (AS): the number of cumulative germinated seeds for each measurement time point, and it was calculated as follows: *N_1_+ (1/2) × N_2_ + (1/3) × N_3_ +…+ (1/T) × N_T_*, which may be proportional to a weighted average of the speed, in which the weight of each element of the equation increases with the measurement time point. This index describes the accumulated number of seed germinated in each measurement period.

Although G_T_ is very useful for the overall assessment of germination (whether there is inhibition, stimulation or whether the compound has no effect on germination), this parameter does not consider the possible delay of germination, as it only considers the final result. Therefore, it is not sensitive enough to study a complex physiological process such as germination. For this reason, each parameter provides different interpretations of the effects, but none of them can accurately illustrate by itself the effects that the compound can induce on germination [49].

Pregerminated seeds of the crops and weeds species were used to conduct growth essays (post-emergence) with the objective of preventing the possible interference of the compound in the germination. The pregerminated seeds were obtained after placing them in trays with filter paper moistened with distilled water and left to grow, under the conditions already mentioned for each species, until their radicle reached 2 mm in length. Five replicates were used per treatment and each replicate had approximately 15–25 seeds, depending on the size of the seed. The treatments were exactly as for the germination bioassays. Once the seeds were placed on the dishes, they were introduced in growth chambers under the same conditions. The test was completed when no more growth of the seedlings was observed. After, the dishes were frozen at −20 °C to prevent growth. The length of the root was measured, and dose–response curves were created to calculate the IC_50_, IC_80_ and LCIC values, which are the NOR concentrations at which 50%, 80% and 100% of the root growth inhibition happens.

#### 5.1.2. Monitoring of *E. bonariensis* Pre-Emergence

A monitoring experiment with *E. bonariensis* was conducted to detect whether the phytotoxic effect of norharmane is stable through the experimental time or experiences relevant variations. *E. bonariensis* was selected due to its growing importance as an invasive species in Spain and its presence in wheat and maize fields, two of the crops studied in the previous bioassays. The tests were conducted in vitro, using different concentrations of norharmane, to control the pre and post-emergence of the weed *E. bonariensis*, which seeds were collected in July 2018 in Carlet (Valencia) in a field of organically produced persimmons, not treated with herbicides.

*E. bonariensis* seeds (20 seeds per replicate) were sown on Petri dishes of 9 cm in diameter between 2 filter paper disks (73 g m^−2^). The filter paper disks were moistened with 5 mL of distilled water treated with the corresponding dose of NOR. The dishes were sealed with Parafilm^®^ and placed in an Equitec germination chamber under conditions of controlled light and temperature for 15 days (needed to obtain a minimum germination of 50% in the control dishes). The conditions inside of the chamber were of 30 °C/16 h of light and 20 °C/8 h of darkness, with 10 replicates per concentration.

The NOR doses used were established using the dose of IC_80_ on *A. thaliana* as a reference, which was previously calculated in experiments conducted by López-González et al. [19], which means that the concentrations tested were IC_80_ (153 µM), IC_80_ × 2 (306 µM), IC_80_ × 4 (612 µM) and IC_80_ × 8 (1224 µM). The concentrations were prepared dissolving NOR in distilled water using 0.05% DMSO as a solvent. Control dishes were also prepared using distilled water and control dishes with 0.05% DMSO. The pH was adjusted to 6.0 to avoid non-desired pH related effects.

The number of germinated seeds and the root length were monitored on days 3, 5, 7, 10 and 14 after incubation, calculating the G_T_ and S indexes and the dose–response curves mentioned in the previous section.

### 5.2. Phytotoxicity Bioassay on the Metabolism of Adult Plants

*A. thaliana* seeds of the Columbia ecotype (Col-0) were sterilized with 50% EtOH and 0.5% NaOCl for 3 min each. After, the seeds were washed with Milli-Q water and were soaked in 0.1% agar. The seeds were at 4 °C for 72 h to promote their vernalization and facilitate the synchronization of germination. After that, the seeds were sown in Petri dishes of 14 cm diameter in a medium of agar with a mixture of macro and micronutrients (Murashige-Skoog, Sigma-Aldrich, Saint Louis, MO, USA), supplemented with 1% sucrose. The seeds were placed on the dish in a concentric pattern leaving 2 cm^3^ per seed. Then, dishes were placed in a growth chamber for 15 days at a temperature of 22 ± 2 °C, a photoperiod of 8 h of light and 16 h of darkness and a relative humidity of 55%.

After 15 days, the seedlings were each transferred to individual 5 cm × 6 cm pots containing Perlite and watering them during 15 days with a 50% Hoagland nutrient solution under the aforementioned environmental conditions, with a light intensity of 140 µmol m^−2^ s^−1^. After this time, the plants entered the adult stage and the treatment with NOR started. 

Four NOR concentrations were used: 0, 153 (IC_80_ in seedling), 306 and 612 µM, prepared in 0.1% DMSO. For each concentration 2 blocks of 6 pots each were used. One of the blocks was irrigated with the compound, whereas the other block was sprayed with the compound, in order to test if the compound was more effective when it was absorbed through the root or was it by contact with the leaves.

For the irrigation treatments, 150 mL of 50% Hoagland solution (pH 6.0) were used and NOR was added at the required concentration dissolved in 0.1% DMSO. Controls were also watered with 50% Hoagland solution and 0.1% DMSO. All the plants were watered every two days during the 21 days that the experiment lasted. 

For the spraying treatments, plants were pulverized with 25 mL of water with a pH of 6.0 with NOR at the required concentration dissolved in 0.1% DMSO. Water with a pH of 6.0 and 0.1% DMSO was used for the control plants. The leaves were pulverized homogenously once a day throughout the 21 days that the experiment lasted. The plants were also watered with 50% Hoagland solution every other day during the whole experiment.

Pictures of the plants were taken every two days during the 21 days of treatment, to observe the changes to their morphology, size and pigmentation.

#### 5.2.1. Measurement of Chlorophyll *a* Fluorescence

The chlorophyll *a* fluorescence of the NOR-treated plants was measured every two days throughout the 21 days of treatment in six whole plants per treatment (the same six along the experiment), according to Graña et al. [26]. Chlorophyll *a* fluorescence was measured using the Maxi-Imaging-PAM Chlorophyll Fluorescence System (Walz, Effeltrich, Germany), which not only provides all parameters related to the measurement of chlorophyll *a* fluorescence, but it also allows to obtain images of such fluorescence in order to get an image of the photosynthetic activity of the whole plant and its spatiotemporal variations [50]. This fluorometer provides parameters related to the photosynthetic capacity through pulse-amplitude-modulation (PAM) on dark-adapted leaves. For this purpose, the plants were kept in darkness for at least 20 min. After this, a very low level of light was applied (0.5 µmol m^−2^ s^−1^) to determine the initial fluorescence (F*_0_*). Subsequently, a high-intensity light pulse (2700 µmol m^−2^ s^−1^) was used to obtain F*m* (the maximum fluorescence of dark-adapted leaves), so the fluorescence level obtained did not include the quantum yield (photochemical quenching). Afterwards, plants were illuminated for 5 min with an actinic light of 110 µmol m^−2^ s^−1^ interrupted every 20 s with high-intensity light saturating pulses of 800 ms (2700 µmol m^−2^ s^−1^) for measuring F*m’*, the maximum fluorescence of light-adapted leaves. The saturation pulses continued and the actinic light enabled the photosynthesis. Therefore, the initial values obtained were compared to the “normal” values (when the photosynthesis is happening), analyzing the photochemical quenching and, by extension, the efficiency of PSII [50]. These measurements were used by the Imaging-PAM to respectively calculate the minimum fluorescence of the light-adapted leaves, F*_0_*’ and the variable fluorescence of the dark-adapted leaves F*v* = F*m* − F_0_ and F*v*’ = F*m*’ − F*_0_*’.

The parameters determined were:

1. Φ_II_ is the the effective photochemical quantum yield of Photosystem II (PSII), which measures the proportion of light absorbed by the chlorophyll associated to PSII that is used in the quantum yield. It can provide insight into the speed of the linear electron flow and, therefore, can be an indicator of the overall photosynthetic capacity in vivo [51]. Φ_II_ + Φ_NPQ_ + Φ_NO_ = 1.
2. Φ_NPQ_ is the measurement of regulated non-photochemical non-fluorescent energy dissipation (as heat). This parameter is linearly related to heat dissipation and ranges from 0 to infinity. The common values in a healthy plant are in the range of 0.5–3.5 at saturating light intensities, although this varies between different species [51]. High values of this parameter indicate an excessive photon flux density, so that plant protects itself dissipating the energy as heat. An increase of this parameter indicates that excess energy is being produced in the photosynthetic apparatus, but the plant is able to regulate such excess avoiding damages; Φ_NPQ_ = 1 − Φ_II_ − Φ_NO_.
3. Φ_NO_ is the measurement of non-regulated emission of energy (as fluorescence). It determines the non-regulated energy loss as fluorescence [52]. High values in this parameter indicate that photochemical energy conversion and protective regulatory mechanisms are inefficient, which means that the plant is struggling to deal with incident radiation. Very high values indicate that the PSII reaction centers are blocked, preventing the build-up of a trans-thylakoid proton gradient, and suggest that the plant has been damaged or is about to be; Φ_NO_ = F*s*/F*m*.
4. F*v*/F*m* is the maximum efficiency of PSII. After being exposed to darkness, PSII reaction centers are normally open (F = F_0_), non-photochemical quenching is minimal (NPQ = 0) and the fluorescence yield is at its maximum. In this state, the increase of fluorescence is induced by saturation pulses (F*v*) and the quantum yield of PSII is the maximum. A reduction of this parameter usually indicates physical damages in the antenna system of PSII; F*v*/F*m* = (F*m* − F_0_)/*Fm*.
5. *ETR* is the apparent electron transport rate. Estimate of the electron flow rate, calculated with the following equation. ETR = 0.5 × Yield × PAR × 0.84 μequiv m^−2^ s^−1^, where PAR is the distance between the sample and the LED lighting unit (Cond. Est. = 18.5 cm).

Fifteen measurements were obtained for each parameter at each measuring time, which yielded a kinetic plot for each parameter along time. The integral value of the area was obtained for all of these graphs. The value represented in the graphs of fluorescence is the average area that was calculated from kinetic measurement for six replicates in each treatment. This area highlights the magnitude of change and can be used to observe the total value of the trend.

#### 5.2.2. Post-Harvest Measurements

After 21 days of treatment, the aerial parts of the plants were harvested in order to measure the following:

##### Measurement of Dry Weight/Fresh Weight Ratio (DW/FW)

The whole aerial parts of three *A. thaliana* plants were used per treatment. They were previously weighted to know their fresh weight (FW), dried with a heater at 70 °C during 72 h and weighted again to know their dry weight (DW). Once these values were obtained, the dry weight/fresh weight (DW/FW) ratio was calculated for the different treatments.

##### Measurement of Osmotic Potential (Ψs)

Fully developed leaves of three independent *A. thaliana* plants for each treatment were used. Leaves were frozen to facilitate the extraction of leaf juice. This parameter was analyzed using the Löser Type 6 osmometer.

##### Measurement of Photosynthetic Pigments

The total content of chlorophyll *a*, chlorophyll *b* and carotenoids was measured and calculated according to Wellburn [53] in three independent biological replicates at the end of the experiment. For each replicate, 100 mg of fresh weight was previously homogenized with liquid N_2_. Subsequently, 1.5 mL of methanol was added to each sample and then centrifuged at 1139 RPM for 5 min. Another 500 µL of methanol were added to 500 µL of supernatant. Lastly, the absorbance of the samples was measured in the spectrophotometer at the following wavelengths: 470, 653, 666 and 750 nm. Once the absorbances were obtained, the total amount of chlorophyll *a*, *b* and carotenoids (xanthophylls and carotenes) was calculated with the Wellburn equations:

Chlorophyll *a* = (15.65 (OD_666_ − OD_750_) − 7.34 (OD_653_ − OD_750_)) V (1)

Chlorophyll *b* = (27.05 (OD_653_ − OD_750_) − 11.21 (OD_666_ − OD_750_)) V (2)

Carotenoids (X + C) = ((1000 (OD_470_ − OD_750_) − 2.86 Chl *a* − 129.9 Chl *b*)/ 221) V (3)

where OD is the optical density at a specific wavelength and V is the methanol volume used (mL). The equations provide the pigment quantity in micrograms. The data were given in micrograms of protein per gram of dry weight and presented as a percentage of the control.

##### Measurement of Anthocyanins

The concentration of anthocyanins was quantified in 3 replicates per treatment, according to the Close method [54] with slight modifications. For each replicate, 1 mL of extraction buffer was added to 100 mg of fresh material, previously homogenized. Subsequently, the samples were centrifuged at 4522 RPM for 20 min, and the absorbance of the supernatant was measured at 530 and 657 nm. The concentration of anthocyanins was obtained with the following formula:

A = (OD_530_ − 0.25 OD_657_) M^−1^ (4)

where M is the amount of plant material used. The data were calculated per gram of dry weight and presented as a percentage of the control.

##### Measurement of Total Proteins

The concentration of total proteins was quantified according to the Bradford method modified by Bonjoch and Tamayo [55] in three biological replicates per treatment. For each replicate, 1 mL of extraction buffer and 50 mg of polyvinylpolypyrrolidone were added to 100 mg of fresh material, previously homogenized. The samples were centrifuged at 4522 RPM for 20 min, and the absorbance of the supernatant was measured at 595 nm. Bovine serum albumin was used as a standard. Protein content was calculated per gram of dry weight and presented as a percentage of the control.

##### Colorimetric Analysis and Leaf Surface

All the leaves from 3 plants per treatment were scanned with the ImageScanner III and the software EPSON EXPRESSION 10000 XL. The images were analyzed with the software Image ProPlus (Media Cybernetic Inc., Bethesda, MD, USA) to obtain the percentage of the areas of the different colors observed (green, yellow, brown or violet), and the total leaf area (calculated as the total number of pixels per image).

### 5.3. Statistical Analysis

After the exclusion of outliers using SPSS Statistics 15.0 and testing for non-normality by the Kolmogorov–Smirnov test and for heteroscedasticity by the Levene’s test, the statistical significance of differences among group means was estimated by ANOVA followed by least significant difference tests for homoscedastic data and by Tamhane’s T2 test for heteroscedastic data. Kruskal–Wallis test was used in the case of non-normally distributed data. SPSS Statistic 22.0 version was used for germination and growth bioassays of crops and weeds and SPSS Statistics 15.0 version was used for the rest of the measurements.

For the statistical analysis of the monitorization of pre-emergence *E. bonariensis* bioassays, the data obtained were processed using the statistical program StatGraphics Centurion XVII. The homoscedasticity of the data was previously tested in order to perform a variance analysis (ANOVA) of all the results. The percentage of inhibition was previously transformed using the formula i = arcsin√x, where x is the percentage of germination expressed on a per unit basis.

The multiple comparison Fisher test (LSD intervals) was used to separate the means, with a confidence level of 95% (*p* ≤ 0.05). The significant differences between the different treatments were expressed with different letters in the same column of the results table.

## Figures and Tables

**Table 1:** Values of G_T_ (total germination rate, expressed as percentage with respect to the control), S (average speed of germination) and AS (speed of accumulated germination) obtained by crop and weed species in germination assays after treatment with NOR concentrations (0–800 µM).

*P. lanceolata*	G_T_ (%)	S	AS	*E. crus-galli*	G_T_ (%)	S	AS	*P. oleracea*	G_T_ (%)	S	AS
Control	100 ^ab^	2.6 ^ab^	7.9 ^a^	Control	100 ^a^	3.48 ^a^	12.55 ^a^	Control	100 ^ab^	3.10 ^ab^	9.63 ^a^
50 µM	115 ^a^	2.9 ^a^	9.2 ^a^	50 µM	101 ^a^	3.62 ^a^	12.56 ^ab^	50 µM	95.4 ^ab^	3.09 ^ab^	9.52 ^a^
100 µM	111 ^ab^	3.4 ^a^	10.2 ^a^	100 µM	98.7 ^a^	3.4o ^a^	10.94 ^ab^	100 µM	136 ^a^	3.80 ^ab^	13.5 ^a^
200 µM	*94.5 ^b^*	2.6 ^a^	7.8 ^a^	200 µM	90.2 ^a^	3.1o ^a^	9.45 ^ab^	200 µM	131 ^ab^	4.20 ^a^	12.9 ^a^
400 µM	97.9 ^ab^	1.8 ^b^	*5.1 ^b^*	400 µM	91.4 ^a^	3.15 ^a^	9.52 ^ab^	400 µM	*76.7 ^b^*	2.57 ^b^	8.00 ^a^
800 µM	*27.1 ^b^*	*1 ^c^*	*1.6 ^c^*	800 µM	90.6 ^a^	3.20 ^a^	*9.57 ^b^*	800 µM	99.5 ^ab^	2.73 ^b^	8.43 ^a^
***A. retroflexus***	**G_T_ (%)**	**S**	**AS**	***A. fatua***	**G_T_ (%)**	**S**	**AS**	***T. aestivum***	**G_T_ (%)**	**S**	**AS**
Control	100 ^a^	5.90 ^ab^	25.9 ^ab^	Control	100 ^ab^	3.74 ^a^	13.0 ^a^	Control	100 ^a^	11.9 ^a^	28.4 ^a^
50 µM	102 ^a^	6.00 ^ab^	26.5 ^ab^	50 µM	104 ^ab^	3.96 ^a^	13.9 ^a^	50 µM	101 ^a^	11.2 ^ab^	28.5 ^a^
100 µM	102 ^a^	6.40 ^a^	27.7 ^ab^	100 µM	92.8 ^a^	3.87 ^a^	13.8 ^a^	100 µM	121 ^a^	10.3 ^ab^	23.5 ^ab^
200 µM	103 ^a^	6.40 ^ab^	28.1 ^a^	200 µM	*76.7 ^bc^*	3.35 ^ab^	12.0 ^ab^	200 µM	128 ^a^	11.0 ^ab^	25.7 ^ab^
400 µM	99.2 ^a^	6.80 ^ab^	29.2 ^ab^	400 µM	81.4 ^abc^	3.10 ^ab^	10.8 ^ab^	400 µM	119 ^a^	10.2 ^ab^	23.7 ^ab^
800 µM	101 ^a^	7.10 ^b^	30.4 ^b^	800 µM	*57.0 ^c^*	*1.88 ^b^*	*6.33 ^b^*	800 µM	90.3 ^a^	*9.36 ^b^*	*21.6 ^b^*
***O. sativa***	**G_T_ (%)**	**S**	**AS**	***Z. mays***	**G_T_ (%)**	**S**	**AS**	***L. sativa***	**G_T_ (%)**	**S**	**AS**
Control	100 ^ab^	4.86 ^abc^	11.4 ^abc^	Control	100 ^ab^	7.96 ^a^	18.6 ^a^	Control	100 ^a^	2.33 ^a^	5.79 ^a^
50 µM	118 ^ab^	6.53 ^a^	15.0 ^a^	50 µM	91.7 ^ab^	6.64 ^ab^	*14.3 ^bc^*	50 µM	84.8 ^ab^	1.96 ^ab^	4.79 ^ab^
100 µM	101 ^ab^	4.90 ^b^	12.0 ^b^	100 µM	81.5 ^ab^	6.45 ^ab^	*14.1 ^bc^*	100 µM	88.4 ^ab^	2.04 ^ab^	5.36 ^ab^
200 µM	115 ^a^	5.40 ^b^	12.3 ^ab^	200 µM	95.7 ^a^	7.93 ^a^	17.1 ^ab^	200 µM	*71.6 ^b^*	*1.64 ^b^*	*4.90 ^b^*
400 µM	*80.8 ^b^*	3.64 ^c^	8.74 ^c^	400 µM	85.2 ^ab^	*5.91 ^b^*	*12.3 ^c^*	400 µM	*41.8 ^c^*	*0.96 ^c^*	*2.29 ^c^*
800 µM	79.6 ^ab^	4.33 ^abc^	10.7 ^abc^	800 µM	82.4 ^b^	*6.00 ^b^*	*14.0 ^bc^*	800 µM	*47.8 ^c^*	*1.10 ^c^*	*2.64 ^c^*

Shaded values are significantly less than those of controls (*p* ≤ 0.05). Numbers within rows followed by same lowercase letters indicate no differences among treatments, while different lowercase letters indicate differences among treatments (*p* ≤ 0.05).

**Table 2:** Values of the root length for weed and crop species after treatment with NOR concentrations of 0–800 µM, expressed as a percentage with respect to the control. The concentrations of NOR that cause 50% and 80% inhibition of seedling growth (IC_50_ and IC_80_) are included below for each target species.

Root Length (%)	*P. lanceolata*	*A. retroflexus*	*A. fatua*	*E. crus-galli*	*P. oleracea*	*Z. mays*	*L. sativa*	*T. aestivum*	*O. sativa*
Control	100 ^a^	100 ^a^	100 ^a^	100 ^a^	100 ^a^	100 ^a^	100 ^a^	100 ^a^	100 ^a^
50 µM	*75.0 ^b^*	*88.9 ^b^*	91.7 ^a^	*85.9 ^b^*	**154 ^b^**	95.5 ^a^	**120 ^b^**	90.3 ^ab^	*93.6 ^b^*
100 µM	*78.5 ^b^*	96.0 ^ac^	105 ^a^	*81.9 ^b^*	**142 ^b^**	106 ^ab^	108 ^ac^	86.6 ^ab^	96.8 ^ab^
200 µM	*68.8 ^b^*	*90.6 ^bc^*	94.7 ^a^	*73.5 ^b^*	102 ^a^	**110 ^b^**	**114 ^bc^**	91.6 ^ab^	101 ^ab^
400 µM	*39.5 ^c^*	*74.0 ^d^*	*59.4 ^b^*	*75.1 ^b^*	*57.1 ^c^*	**111 ^b^**	*67.3 ^d^*	*74.9 ^b^*	99.0 ^ab^
800 µM	*8.46 ^d^*	*56.6 ^e^*	*19.8 ^c^*	*49.4 ^c^*	*9.85 ^d^*	98.0 ^ab^	*38.8 ^e^*	*51.3 ^c^*	*87.7 ^c^*
IC_50_	353 µM	890 µM	525 µM	767 µM	511 µM	Tolerant	893 µM	Out of range	Tolerant
IC_80_	661 µM	Out of range	809 µM	Out of range	713 µM	Tolerant	Out of range	Out of range	Tolerant

Italicized values are significantly less and bold values are significantly greater than the control (*p* ≤ 0.05). Numbers within rows followed by same lowercase letters indicate no differences among treatments, while different lowercase letters indicate differences among treatments (*p* ≤ 0.05).

**Table 3:** Crops and associated weeds tested in this work.

Crop	Associated Weed
*Zea mays* (maize)	*Echinochloa crus-galli* (barnyard grass),
	*Amaranthus retroflexus* (redroot pigweed)
	*Portulaca oleracea* (common purslane)
	*Erigeron bonariensis* (flax-leaf fleabane)
*Triticum aestivum* (wheat)	*Avena fatua* (wild oat)
	*Plantago lanceolata* (ribwort)
	*Erigeron bonariensis* (flax-leaf fleabane)
*Oryza sativa* (rice)	*Echinochloa crus-galli* (barnyard grass)
	*Portulaca oleracea* (common purslane)
*Lactuca sativa* (lettuce)	*Portulaca oleracea* (common purslane)
	*Plantago lanceolata* (ribwort)
